# Association Between Healthful Plant‐Based Dietary Pattern and Adiposity Measures Trajectories and Future Metabolic Diseases Risk: A Prospective Cohort Study

**DOI:** 10.1002/fsn3.70790

**Published:** 2025-08-17

**Authors:** Chenyu Zhao, Tianrun Wang, Yuping Wang, Xiaocan Jia, Zhixing Fan, Chaojun Yang, Jingwen Fan, Nana Wang, Yongli Yang, Xuezhong Shi, Yifan Shan

**Affiliations:** ^1^ Department of Epidemiology and Biostatistics, College of Public Health Zhengzhou University Zhengzhou Henan China; ^2^ School of Public Health Jilin University Changchun Jilin China; ^3^ Mental Health Education Center Zhengzhou University Zhengzhou Henan China; ^4^ Department of Cardiology, The First College of Clinical Medical Science, China Three Gorges University and Yichang Central People's Hospital Yichang Hubei China; ^5^ Institute for Hospital Management of Henan Province, The First Affiliated Hospital of Zhengzhou University Zhengzhou Henan China

**Keywords:** adiposity measures trajectory, cohort study, metabolic diseases, plant‐based dietary pattern

## Abstract

The dynamic and heterogeneous process of obesity measurement can be better assessed by change trajectories. Utilizing multiple metrics to assess obesity could provide more comprehensive insights. Currently, the associations of adiposity measures trajectories with metabolic diseases and plant‐based dietary patterns remain unclear. Using latent class mixed modeling approach, we identified body mass index (BMI), waist‐to‐hip ratio (WHR) and fat mass index (FMI) trajectory groups based on measures acquired at four time points. We examined associations between adiposity measures trajectories and plant‐based dietary patterns, using logistic regression. Cox proportional hazards regression models were applied to investigate the association between adiposity measures trajectories and metabolic diseases. We identified two latent classes of BMI trajectories: low‐smooth and high‐growth‐decline, two WHR trajectories: low‐growth and high‐growth, and two FMI trajectories: low‐smooth and high‐growth‐decline. Participants who had a high healthful plant‐based diet index had lower odds of being in the high‐growth‐decline BMI trajectory (OR = 0.491, 95% CI: 0.402, 0.600), the high‐growth WHR trajectory (OR = 0.526, 95% CI: 0.438, 0.632) or the high‐growth‐decline FMI trajectory (OR = 0.533, 95% CI: 0.446, 0.638). We found that participants in the high‐growth‐decline BMI trajectory (HR = 1.925, 95% CI: 1.542, 2.404), the high‐growth WHR trajectory (HR = 1.314, 95% CI: 1.003, 1.722) or the high‐growth‐decline FMI trajectory (HR = 1.562, 95% CI: 1.236, 1.975) had higher risks. A healthy plant‐based dietary pattern assists in maintaining normal body size over time. Concurrently, long‐term stabilization of a normal body size may be linked to a reduced risk of metabolic diseases.

## Graphical abstract

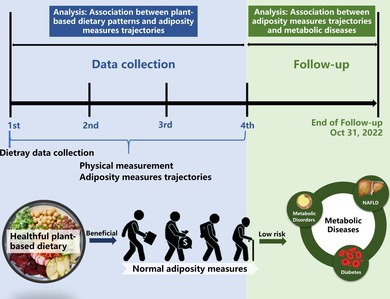

## 1 Introduction

Over the past five decades, obesity has increased steadily, reaching pandemic levels globally (Blüher 2019; Perdomo et al. 2023). Obesity, based on Body Mass Index (BMI) measurements, constitutes a significant risk factor for various chronic illnesses, encompassing cardiovascular diseases, numerous cancers, and metabolic disorders (Collaborators et al. 2017). Given its high prevalence and associated health burden, obesity remains a critical global public health challenge.

Most evidence linking adiposity to metabolic disease has been derived from epidemiological studies using BMI as the primary measure (Booth et al. 2015; Simchoni et al. 2020; Wang, Yi, et al. 2023). Although BMI is widely used, it does not capture fat distribution or body composition. Alternative measures such as Fat Mass Index (FMI) and waist‐to‐hip ratio (WHR) have shown stronger associations with disease incidence and mortality in observational studies (Czernichow et al. 2011; Emdin et al. 2017; Liu et al. 2013). These metrics may complement or outperform BMI in estimating the risk of adiposity‐related metabolic diseases. Given the dynamic and heterogeneous nature of body fat accumulation, repeated assessments over time may offer a more accurate understanding of adiposity‐related risks. Identifying trajectories of adiposity could inform early prevention strategies and improve risk stratification. However, longitudinal analyses examining these trajectories and their links to metabolic disease remain limited.

Diet plays a key role in metabolic health (Kitada et al. 2019). In particular, plant‐based diets have been recognized as modifiable lifestyle factors that reduce the risk of chronic diseases (Martin et al. 2013; Wang, Masedunskas, et al. 2023). A growing body of evidence supports the protective effects of plant‐based diets against obesity and metabolic disorders (Chen, Zeng, et al. 2022; Muscogiuri et al. 2022; Wang et al. 2021). Nonetheless, prior research has largely relied on cross‐sectional data, limiting insight into the long‐term influence of plant‐based diets on adiposity patterns. The relationship between sustained plant‐based dietary habits and longitudinal changes in adiposity remains understudied.

In this study, using data from the UK Biobank, we identified adiposity trajectories based on BMI, WHR, and FMI. We then assessed their associations with metabolic diseases and examined how adherence to plant‐based dietary patterns influenced these adiposity trajectories.

## 2 Materials and Methods

### 2.1 Study Design and Participants

The study population was derived from the UK Biobank study, a large‐scale prospective cohort study. Between 2006 and 2010, participants completed a comprehensive baseline assessment that included self‐reported data on well‐being, lifestyle, behavior, and medical history, along with physical measurements and biological sample collection at centers across the UK. Follow‐up assessments were subsequently conducted in 2012, 2014, and 2019. Detailed information regarding the study's design, methodologies, and rationale for the UK Biobank cohort has been previously published (Sudlow et al. 2015). Ethical approval for the UK Biobank research was obtained from the North West Multicenter Research Ethics Committee, and written informed consent was obtained from all participants prior to their participation in the study.

To construct adiposity trajectories, we initially included 12,678 participants who were enrolled between 2006 and 2011 and who had physical measurement data available at least three of the four time points. We then excluded 4959 participants who discontinued their participation in the study, had incomplete data on food intake, or exhibited metabolic diseases at the final physical measurement, resulting in a cohort of 7719 participants for analysis (Figure S1).

### 2.2 Adiposity Measures Trajectories Assessment

Adiposity measures encompassed BMI, WHR, and FMI, calculated from anthropometric data including weight and height, waist and hip circumference, and bioelectrical impedance analysis (BIA), respectively. FMI represents the proportion of fat mass (measured in kilograms) to height (measured in meters). FMI was employed as an alternative to whole‐body fat mass to standardize for height, providing an absolute measure of fat mass‐specific adiposity. WHR serves as a dimensionless proxy for abdominal adiposity.

Distinct trajectories of BMI, WHR, or FMI were identified using a latent class mixed modeling (LCMM) approach (Lennon et al. 2018), which utilized adiposity data from the four time points. The analysis was conducted using the “lcmm” package within the R software environment (Proust‐Lima et al. 2017) (see Supporting Information for further details).

### 2.3 Dietary Assessment and Calculation of Plant‐Based Diet Indices

Dietary data were obtained through a 24‐h dietary recall questionnaire (Liu et al. 2011). The questionnaire's specifics have been previously outlined and validated for estimating similar nutrient intakes based on a single day's dietary intake (Piernas et al. 2021). We derived the plant‐based diet index (PDI), healthful plant‐based diet index (hPDI), and unhealthful plant‐based diet index (uPDI) by assigning scores to 17 food categories, following established methods (Thompson et al. 2023) (see Supporting Information for further details). Each participant underwent multiple dietary assessments during the follow‐up period. According to previous research, there is a strong correlation between repeated dietary assessments (Heianza et al. 2021). Therefore, for participants who completed more than one dietary evaluation, the earliest dietary intake data were used in this study to maximize the length of follow‐up.

The final PDI, hPDI, and uPDI scores for each participant were calculated by summing the scores from each of the 17 food groups. Subsequently, PDI, hPDI, and uPDI were categorized into three groups: low (< P25), medium (P25–P75), and high (≥ P75).

### 2.4 Metabolic Diseases Ascertainment

The outcomes of interest are the metabolic diseases, including non‐alcoholic fatty liver disease (NAFLD), diabetes, and broader metabolic disorders (e.g., disorders of lipoprotein metabolism, electrolyte metabolism disorders, etc.) (Chew et al. 2023; Chong et al. 2023). Outcomes were defined based on the International Statistical Classification of Diseases and Related Health Problems, Tenth Revision (ICD‐10) codes (Table S1). We calculated the follow‐up time from the date of the final physical examination to the date of metabolic diseases or October 31, 2022, whichever came first.

### 2.5 Covariates

To mitigate potential confounding factors, we integrated several covariates encompassing sociodemographic characteristics such as age, gender, ethnicity, educational attainment, Townsend deprivation index (TDI), as well as lifestyle variables including smoking, alcohol consumption, and physical activity. Further intricate insights into the gathered data and measurements can be found in previous studies. These covariates were extrapolated from the baseline survey.

### 2.6 Statistical Analysis

To manage partial missing data in covariates, we employed an imputation approach utilizing chained equations to produce a comprehensive dataset.

The logistic regression model was employed to investigate the association between groups of PDI, hPDI, or uPDI and trajectories of adiposity measures. It was adjusted for age, sex, ethnicity, educational level, TDI, smoking, drinking, and physical activity. Trend tests were conducted by assigning the median value to each group of an index and entering it as a continuous variable in the model. Additionally, we treated these indices as continuous variables (per SD‐unit increment). Furthermore, we evaluated the dose–response relationship between hPDI and adiposity measures using restricted cubic spline regression. We chose the top two covariates with effects in the model, which test the relationship between hPDI and trajectories of adiposity measures, for subgroup analyses.

Associations between trajectories of adiposity measures and metabolic diseases were examined using Cox proportional hazards analysis. A two‐stage modeling process was employed. Model 1 did not include adjustment covariates, while Model 2 additionally adjusted for age, sex, ethnicity, educational level, TDI, smoking, drinking, and physical activity. The assumption of proportional hazards was assessed using Schoenfeld residuals, and no violations were observed. Subsequently, Kaplan–Meier survival curves and log‐rank tests were utilized to assess the incidence of metabolic diseases across adiposity measures trajectories in all participants. To ensure the robustness of the results, several sensitivity analyses were performed. Initially, participants with less than 2 years of follow‐up were excluded. Secondly, the Fine–Gray competing‐risk model was employed to estimate hazard ratios (HRs) for comparison. Finally, we excluded participants with missing covariates.

Statistical analyses were done using SAS (version 9.4) and all tests were two‐sided with *p* < 0.05 indicating statistical significance.

## 3 Results

### 3.1 Baseline Characteristics

Table 1 shows the baseline characteristics of participants. During a median of 3.39 years of follow‐up, 512 cases of metabolic diseases were identified. Participants with metabolic diseases were more likely to be male, have a low level of education, smoke, drink, and have low physical activity compared with those without metabolic diseases.

**TABLE 1:** Baseline characteristics of participants included.

Variables	Overall (*n* = 7719)	Without metabolic diseases (*n* = 7207)	With metabolic diseases (*n* = 512)
Age (mean (SD))	54.71 (7.48)	54.45 (7.48)	58.37 (6.54)
Sex (%)			
Female	4026 (52.2)	3830 (53.1)	196 (38.3)
Male	3693 (47.8)	3377 (46.9)	316 (61.7)
Ethnic (%)			
White	7523 (97.5)	7021 (97.4)	502 (98.0)
Others	183 (2.4)	173 (2.4)	10 (2.0)
Missing	13 (0.2)	13 (0.2)	0 (0.0)
Qualifications (%)			
College or University degree	4002 (51.8)	3773 (52.4)	229 (44.7)
A levels/AS levels or equivalent	1089 (14.1)	1016 (14.1)	73 (14.3)
O levels/GCSEs or equivalent	1372 (17.8)	1279 (17.7)	93 (18.2)
Professional qualifications	346 (4.5)	317 (4.4)	29 (5.7)
None of the above	895 (11.6)	808 (11.2)	87 (17.0)
Missing	15 (0.2)	14 (0.2)	1 (0.2)
TDI (mean (SD))	−2.15 (2.56)	−2.16 (2.55)	−2.07 (2.66)
Smoking (%)			
Never	4841 (62.7)	4566 (63.4)	275 (53.7)
Previous	2482 (32.2)	2281 (31.6)	201 (39.3)
Current	385 (5.0)	351 (4.9)	34 (6.6)
Missing	11 (0.1)	9 (0.1)	2 (0.4)
Drinking (%)			
Never	201 (2.6)	190 (2.6)	11 (2.1)
Previous	144 (1.9)	132 (1.8)	12 (2.3)
Current	7373 (95.5)	6884 (95.5)	489 (95.5)
Missing	1 (0.0)	1 (0.0)	0 (0.0)
Physical activity (%)			
Low	1182 (15.3)	1100 (15.3)	82 (16.0)
Moderate	2797 (36.2)	2615 (36.3)	182 (35.5)
High	2668 (34.6)	2515 (34.9)	153 (29.9)
Missing	1072 (13.9)	977 (13.6)	95 (18.6)

Abbreviations: SD, standard deviation; TDI, Townsend Deprivation Index.

### 3.2 Estimated Adiposity Measures Trajectories Modeling

**FIGURE 1:**
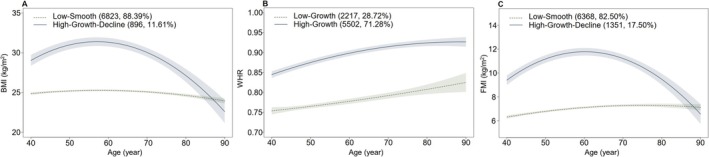
Trajectories of BMI (A), WHR (B), and FMI (C).

We fitted both two‐trajectory and three‐trajectory models for BMI, WHR, and FMI (see Table 2 for details). The average posterior probabilities (APP) for all models exceeded 0.7. For BMI and WHR, the two‐trajectory models (BIC_BMI_ = 101,114.9; BIC_WHR_ = −67,121.8) had Bayesian Information Criterion (BIC) values closer to zero than the three‐trajectory models (BIC_BMI_ = 101,353.8; BIC_WHR_ = −67,127.9), and the proportion of participants in each subgroup of the two‐trajectory models exceeded 5% of the total sample size. In contrast, for FMI, although the BIC of the three‐trajectory model (FMI_BMI_ = 89,313.54) was closer to zero than that of the two‐trajectory model (FMI _BMI_ = 89,772.0), the proportion of participants in one subgroup of the three‐trajectory model was less than 5%. Therefore, the two‐trajectory model was selected as the optimal model for BMI, WHR, and FMI in this study. The best‐fitting model identified two trajectories for BMI, WHR, and FMI, respectively (Figure 1). On the basis of adiposity measures at baseline and growth and decline trend, BMI trajectories were given the following labels: low‐smooth (6823 [88.39%] participants) and high‐growth‐decline (896 [11.61%] participants); WHR trajectories were given the following labels: low‐growth (2217 [28.72%] participants) and high‐growth (5502 [71.28%] participants); FMI trajectories were given the following labels: low‐smooth (6368 [82.50%] participants) and high‐growth‐decline (1351 [17.50%] participants). Table S2 shows the baseline characteristics of participants in trajectory groups.

**TABLE 2:** Fitting statistics for trajectories.

	Number of BMI trajectory						Number of WHR trajectory						Number of FMI trajectory					
	2			3			2			3			2			3		
BIC^a^	101,114.9			101,353.8			−67,121.8			−67,127.9			89,772.0			89,313.54		
Class 1 (*n*/%^b^/APP^c^)	6823	88.39	0.916	415	5.38	0.811	2217	28.72	0.761	2313	29.97	0.739	6368	82.50	0.897	5114	66.25	0.846
Class 2 (*n*/%^b^/APP^c^)	896	11.61	0.846	7147	92.59	0.977	5502	71.28	0.923	101	1.31	0.514	1351	17.50	0.855	2268	29.38	0.767
Class 3 (*n*/%^b^/APP^c^)	/	/	/	157	2.03	0.856	/	/	/	5305	68.73	0.895	/	/	/	337	4.37	0.836

Abbreviations: APP, average posterior probabilities; BIC, Bayesian information criteria; BMI, Body Mass Index; FMI, Fat Mass Index; WHR, waist‐to‐hip ratio.

^a^ The lower absolute value shows a better model fit.

^b^ More than 5% of individuals belonging to each trajectory group is better.

^c^ The higher value is better (> 0.7 in a class).

### 3.3 Adiposity Measures Trajectories and Metabolic Diseases

Based on the Kaplan–Meier curve, differential risks of metabolic diseases were observed for each BMI trajectory (Figure S2A) and WHR trajectory (Figure S2B).

Table 3 presents the outcomes derived from Cox proportional‐hazards models. Following adjustment for covariates, individuals following a high‐growth‐decline BMI trajectory exhibited a 1.925‐fold higher risk of metabolic diseases (HR = 1.925, 95% CI: 1.542, 2.404) compared to those following a low‐smooth BMI trajectory during the follow‐up period. Similarly, individuals following a high‐growth WHR trajectory had a 1.314‐fold higher risk (HR = 1.314, 95% CI: 1.003, 1.722) of metabolic diseases compared to those following a low‐growth WHR trajectory. Furthermore, individuals following a high‐growth‐decline FMI trajectory had a 1.562‐fold higher risk (HR = 1.562, 95% CI: 1.236, 1.975) of metabolic diseases compared to those following a low‐smooth FMI trajectory.

**TABLE 3:** Cox proportional‐hazards models for adiposity measures trajectories and metabolic diseases.

	BMI HR (95% CI)		WHR HR (95% CI)		FMI HR (95% CI)	
	Low‐smooth (*n* = 6823)	High‐growth‐decline (*n* = 896)	Low‐growth (*n* = 2217)	High‐growth (*n* = 5502)	Low‐smooth (*n* = 6368)	High‐growth‐decline (*n* = 1351)
Metabolic diseases						
Cases, *n* (%)	413 (6.05)	99 (11.05)	100 (4.51)	412 (7.49)	407 (6.39)	105 (7.77)
Model 1	Ref	1.916 (1.539, 2.386)	Ref	1.799 (1.445, 2.238)	Ref	1.205 (0.972, 1.493)
Model 2	Ref	1.925 (1.542, 2.404)	Ref	1.314 (1.003, 1.722)	Ref	1.562 (1.236, 1.975)
NAFLD						
Cases, *n* (%)	31 (0.45)	12 (1.34)	5 (0.23)	38 (0.69)	29 (0.46)	14 (1.04)
Model 1	Ref	3.021 (1.552, 5.883)	Ref	3.223 (1.269, 8.188)	Ref	2.249 (1.188, 4.256)
Model 2	Ref	3.030 (1.538, 5.972)	Ref	3.047 (1.084, 8.567)	Ref	3.135 (1.534, 6.406)
Diabetes						
Cases, *n* (%)	50 (0.73)	40 (4.46)	8 (0.36)	82 (1.49)	55 (0.86)	35 (2.59)
Model 1	Ref	6.351 (4.190, 9.625)	Ref	4.358 (2.109, 9.008)	Ref	2.990 (1.957, 4.567)
Model 2	Ref	6.080 (3.973, 9.303)	Ref	3.032 (1.347, 6.823)	Ref	5.037 (3.119, 8.136)
Metabolic disorders						
Cases, *n* (%)	366 (5.36)	68 (7.59)	93 (4.19)	341 (6.20)	355 (5.57)	79 (5.85)
Model 1	Ref	1.463 (1.130, 1.895)	Ref	1.590 (1.264, 1.999)	Ref	1.032 (0.808, 1.317)
Model 2	Ref	1.476 (1.137, 1.917)	Ref	1.139 (0.852, 1.522)	Ref	1.292 (0.993, 1.681)

*Note:* Model 1 had no adjustment covariates; Model 2 was adjusted for age, sex, ethnicity, Townsend deprivation index, educational level, smoking, drinking and physical activity. Abbreviations: BMI, Body Mass Index; CI, Confidence interval; FMI, Fat Mass Index; HR, hazard ratio; NAFLD, Nonalcoholic fatty liver disease; WHR, waist‐to‐hip ratio.

Regarding NAFLD, individuals following a high‐growth‐decline BMI trajectory had a 3.030‐fold higher risk (HR = 3.030, 95% CI: 1.538, 5.972) than those following a low‐smooth BMI trajectory. Similarly, individuals following a high‐growth WHR trajectory had a 3.047‐fold higher risk (HR = 3.047, 95% CI: 1.084, 8.567) than those following a low‐growth WHR trajectory. Additionally, individuals following a high‐growth‐decline FMI trajectory had a 3.135‐fold higher risk (HR = 3.135, 95% CI: 1.534, 6.406) than those following a low‐smooth FMI trajectory.

Concerning diabetes, individuals following a high‐growth‐decline BMI trajectory had a 6.080‐fold higher risk (HR = 6.080, 95% CI: 3.973, 9.303) than those following a low‐smooth BMI trajectory. Similarly, individuals following a high‐growth WHR trajectory had a 3.032‐fold higher risk (HR = 3.032, 95% CI: 1.347, 6.823) than those following a low‐growth WHR trajectory. Moreover, individuals following a high‐growth‐decline FMI trajectory had a 5.037‐fold higher risk (HR = 5.037, 95% CI: 3.119, 8.136) than those following a low‐smooth FMI trajectory.

Concerning metabolic disorders, individuals following a high‐growth‐decline BMI trajectory had a 1.476‐fold higher risk (HR = 1.476, 95% CI: 1.137, 1.917) than those following a low‐smooth BMI trajectory.

The results of the sensitivity analysis were consistent with those of the main analysis (Tables S3–S6).

### 3.4 Healthful Plantbased Dietary Patterns and Adiposity Measures Trajectories

The associations between plant‐based dietary patterns and accelerated adiposity measure trajectories are presented in Table 4. The likelihood of belonging to the high‐growth‐decline BMI trajectory was 50.9% lower (OR = 0.491, 95% CI: 0.402, 0.600) for individuals in the high group and 31.6% lower (OR = 0.684, 95% CI: 0.578, 0.810) for those in the medium group of the hPDI, compared to participants in the low hPDI group. With each standard deviation increase in the hPDI score, the probability of being in the high‐growth‐decline BMI trajectory decreased by 23.1% (OR = 0.769, 95% CI: 0.714, 0.828).

**TABLE 4:** Logistic regression models for plant‐based dietary patterns and adiposity measures trajectories.

Diet Index	Median of Diet Index	High‐growth‐decline BMI trajectory		High‐growth WHR trajectory		FMI high‐growth‐decline trajectory	
		OR (95% CI)	*p*‐trend	OR (95% CI)	*p*‐trend	OR (95% CI)	*p*‐trend
Group of PDI			0.01		0.36		0.04
Low	49	Ref		Ref		Ref	
Medium	55	0.787 (0.665, 0.932)		0.952 (0.814, 1.114)		0.846 (0.724, 0.988)	
High	60	0.779 (0.643, 0.944)		0.922 (0.777, 1.095)		0.830 (0.698, 0.987)	
Per SD, unit increment of PDI		0.915 (0.852, 0.982)		0.969 (0.911, 1.030)		0.938 (0.881, 0.999)	
Group of hPDI			< 0.001		< 0.001		< 0.001
Low	43	Ref		Ref		Ref	
Medium	49	0.684 (0.578, 0.810)		0.717 (0.602, 0.855)		0.697 (0.593, 0.820)	
High	55	0.491 (0.402, 0.600)		0.526 (0.438, 0.632)		0.533 (0.446, 0.638)	
Per SD, unit increment of hPDI		0.769 (0.714, 0.828)		0.768 (0.720, 0.818)		0.790 (0.740, 0.844)	
Group of uPDI			0.75		0.01		0.24
Low	43	Ref		Ref		Ref	
Medium	49	0.980 (0.817, 1.175)		1.030 (0.884, 1.201)		0.983 (0.837, 1.153)	
High	55	1.028 (0.842, 1.255)		1.250 (1.050, 1.489)		1.105 (0.925, 1.320)	
Per SD, unit increment of uPDI		1.042 (0.970, 1.119)		1.105 (1.038, 1.176)		1.046 (0.981, 1.115)	

*Note:* Logistic regression model was adjusted for age, sex, ethnicity, Townsend deprivation index, educational level, smoking, drinking and physical activity. Abbreviations: BMI, Body Mass Index; CI, confidence interval; FMI, Fat Mass Index; hPDI, healthful plant‐based diet index; OR, odds ratio; PDI, Plant‐Based Diet Index; SD, standard deviation; uPDI, unhealthful Plant‐Based Diet Index; WHR, waist‐to‐hip ratio.

Likewise, the probability of being in the high‐growth WHR trajectory was 47.4% lower (OR = 0.526, 95% CI: 0.438, 0.632) for individuals in the high group and 28.3% lower (OR = 0.717, 95% CI: 0.602, 0.855) for those in the medium group of the hPDI compared to participants in the low hPDI group. A 23.2% decrease (OR = 0.768, 95% CI: 0.720, 0.818) in the probability of being in the high‐growth WHR trajectory was observed with each standard deviation increase in the hPDI score.

Furthermore, the probability of being in the high‐growth‐decline FMI trajectory was 46.7% lower (OR = 0.533, 95% CI: 0.446, 0.638) for individuals in the high group and 30.3% lower (OR = 0.697, 95% CI: 0.593, 0.820) for those in the medium group of the hPDI compared to participants in the low hPDI group. A decrease of 21.0% (OR = 0.790, 95% CI: 0.740, 0.844) in the probability of being in the high‐growth‐decline FMI trajectory was observed with each standard deviation increase in the hPDI score.

**FIGURE 2:**
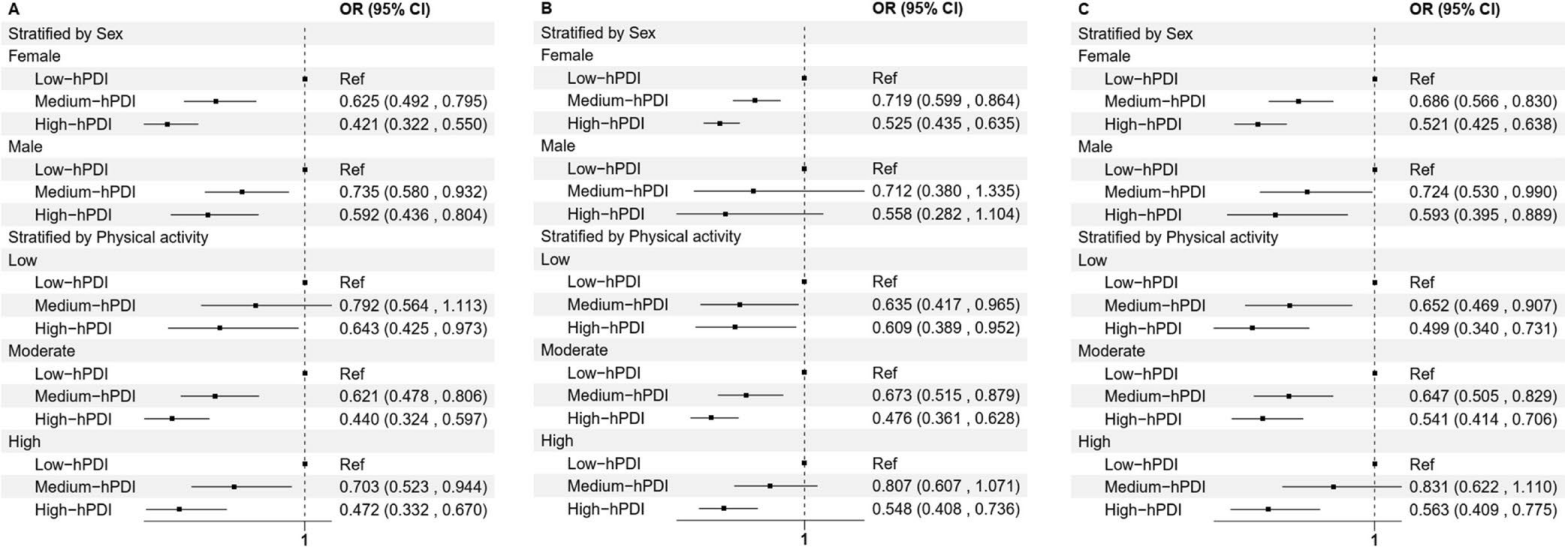
Sex‐ and physical activity‐stratified analyses on the hPDI and adiposity measures trajectories. Model was adjusted for age, ethnicity, Townsend deprivation index, qualifications, smoking, drinking, and physical activity in sex‐stratified analyses; or was adjusted for age, sex, ethnicity, Townsend deprivation index, qualifications, smoking, and drinking in physical activity‐stratified analyses.

Nonlinear correlations between hPDI scores and the high‐growth‐decline BMI, high‐growth WHR, and high‐growth‐decline FMI trajectories were not found to be significant (Figure S3). Sex‐ and physical activity‐stratified analyses were further conducted (Figure 2), demonstrating consistency with the main results.

## 4 Discussion

In this prospective cohort study, we identified two distinct trajectories for each adiposity measure based on repeated assessments over a 10‐year period. Compared to the low‐smooth BMI trajectory, low‐growth WHR trajectory, or the low‐smooth FMI trajectory, individuals following the high‐growth‐decline BMI trajectory, high‐growth WHR trajectory, or high‐growth‐decline FMI trajectory exhibited significantly increased risks of developing metabolic diseases. Moreover, greater adherence to a healthful plant‐based dietary pattern was associated with lower odds of belonging to high‐risk adiposity trajectories.

The trajectories of BMI, WHR, and FMI effectively identified individuals at elevated risk of metabolic diseases. Traditionally, BMI has been the primary metric for assessing overweight and obesity at both population and individual levels, providing a general estimate of total body fat. FMI, determined via BIA, computes each participant's body fat mass and normalizes it by height squared (VanItallie et al. 1990; Wells 2001), thus offering a valuable measure of obesity (Kyle et al. 2003; Liu et al. 2013). In our study, BMI and FMI trajectories demonstrated similar trends, likely reflecting that increases in body fat primarily drive weight gain in most individuals. However, in populations with greater muscle mass, such as athletes. Given the expense and logistical challenges of BIA for FMI measurement (Achamrah et al. 2018), BMI remains a practical tool for routine obesity assessment. Interestingly, a larger proportion of participants were classified into high‐risk trajectory groups by WHR compared to BMI, indicating a stronger upward trend in central adiposity. WHR, as a measure of abdominal fat distribution, can increase even when total body weight remains stable. Previous studies have emphasized the heterogeneity of obesity as defined by BMI, demonstrating that individuals with similar BMI values may have markedly different metabolic profiles and risks (González‐Muniesa et al. 2017; Tchernof and Després 2013). Additionally, WHR has been shown to correlate more strongly with adverse health outcomes than BMI (Harris 2023; Khan et al. 2023). This suggests that individuals within the low‐smooth BMI trajectory may still be at elevated risk if they exhibit increasing WHR over time. Our findings therefore support the clinical utility of monitoring both BMI and WHR trajectories, as their combined use provides a more nuanced assessment of metabolic disease risk.

For high‐risk trajectories—such as high‐growth‐decline BMI trajectory and high‐growth‐decline FMI trajectory—indicate that individuals start at overweight levels, with BMI or FMI continually rising steadily until about age 60. Although these measures subsequently decline to within normal ranges, the detrimental effects of earlier obesity appear to persist. This may be because the decline occurs in later life, a period marked by reduced physiological resilience and diminished response to interventions (Evans et al. 1997). In the high‐growth WHR trajectory, even after BMI and FMI decline after age 60, WHR continues to rise, indicating progressive central fat accumulation. This is particularly concerning, as central adiposity is strongly linked to increased risk of metabolic diseases (Ibrahim 2009). These findings underscore that the adverse effects of early obesity are long‐lasting and not easily reversed, even with later improvements in body weight. Our results highlight the importance of early weight management and sustained efforts to maintain a healthy body composition throughout life, especially regarding the prevention of central fat accumulation.

Diet is one of the primary modifiable lifestyle factors, and a predominantly plant‐based dietary pattern can reduce the risk of obesity (Choi et al. 2020; Damigou et al. 2024). Unlike previous studies that considered only food types and intake frequency (Chen, Shen, et al. 2022; Ren et al. 2023), we also accounted for intake quantity, enabling a more accurate assessment of dietary pattern. Recognizing that not all plant‐based foods are equally beneficial, we used the hPDI to focus on plant‐based foods associated with improved health outcomes (Wang et al. 2021). Our research further demonstrates that a healthy plant‐based dietary pattern can help maintain favorable adiposity trajectories over time. This may be partly explained by the high dietary fiber content of these foods. Fibers such as inulin and oligosaccharides act as prebiotics, influencing gut microbiota, inflammation, insulin sensitivity, and lipid metabolism (Gibson et al. 2017). Specific plant‐derived peptides can inhibit digestive enzymes, such as pancreatic lipase and α‐glucosidase, affecting nutrient absorption and weight management (Carvalho et al. 2025; Wei et al. 2024). Additionally, from a nutrigenomic perspective, genetic variations may affect dietary impacts on gene expression via epigenetic mechanisms such as DNA methylation and histone modifications, influencing metabolic pathways (Ordovas et al. 2018). Integrating genetic mapping and nutrigenomic research into dietary interventions may enhance precision in public health recommendations. However, biases related to genetic predispositions must be considered, as genes such as FTO, MC4R, PPARG, and TMEM18 are associated with obesity and metabolic disorders, especially in European populations (Frayling et al. 2007; Loos et al. 2008). Unfortunately, due to data availability limitations, this study was unable to obtain information on participants' family history of metabolic diseases or genetic risk scores. Future research is needed to account for the potential confounding effects of genetic factors on the associations among diet, obesity, and metabolic diseases.

Our study has several strengths. We used a prospective design with extended follow‐up and assessed adiposity at four time points, allowing us to capture dynamic changes. Dietary patterns were evaluated using a comprehensive approach that considered food type, frequency, and quantity.

Nonetheless, some limitations remain. Firstly, we acknowledge that unmeasured factors such as medication use and baseline comorbidities may influence both diet and adiposity. Certain medications (e.g., glucocorticoids, antipsychotics) are associated with weight gain and may alter appetite or food choices. Similarly, chronic conditions like diabetes or depression can affect both dietary behavior and fat accumulation. While we adjusted for several confounders, the lack of detailed data on these variables is a limitation. Future studies should incorporate comprehensive clinical information to better account for these potential influences and strengthen causal interpretations. Secondly, a potential limitation of our study relates to regression dilution bias, arising from within‐person variability in dietary intake. Inherent fluctuations in participants' daily diets may lead to misclassification and attenuation of true associations. Regression dilution bias can underestimate effect sizes, especially when exposure (dietary intake) is measured with error. However, previous research showed that the PDI derived from a single baseline measure was strongly correlated with the averaged PDI calculated across multiple time points (Heianza et al. 2021), suggesting that the impact of this bias may be modest in our study. Nevertheless, caution is warranted in interpreting effect sizes, and future studies with more frequent or objective dietary assessments could further minimize this source of bias. Third, the UK Biobank may not fully represent the general population and could exhibit selection bias toward healthier volunteers. Lastly, as our participants were predominantly of European descent, the generalizability of our findings to other populations is limited.

## 5 Conclusion

In conclusion, we constructed adiposity measures trajectories using BMI, WHR, and FMI, respectively; evaluated the long‐term dynamic trends of adiposity measures; and found that high‐growth‐decline BMI trajectory, high‐growth WHR trajectory, and high‐growth‐decline FMI trajectory were associated with the risk of developing metabolic diseases, and that healthy plant‐based dietary patterns could reduce the probability of being high‐growth‐decline BMI trajectory or FMI trajectory, or high‐growth WHR trajectory. Our results suggest that increasing healthy plant‐based dietary intake may reduce the long‐term risk of obesity, and that maintaining obesity measures at a normal level over time may reduce the risk of metabolic disease.

## Author Contributions

**Chenyu Zhao:** methodology (lead), writing – original draft (lead). **Tianrun Wang:** methodology (supporting). **Yuping Wang:** methodology (supporting). **Xiaocan Jia:** methodology (supporting). **Zhixing Fan:** data curation (equal), formal analysis (equal). **Chaojun Yang:** writing – review and editing (equal). **Jingwen Fan:** writing – review and editing (equal). **Nana Wang:** writing – review and editing (equal). **Yongli Yang:** conceptualization (supporting), visualization (supporting). **Xuezhong Shi:** conceptualization (lead), resources (lead), supervision (lead), visualization (supporting). **Yifan Shan:** funding acquisition (lead), validation (lead), writing – review and editing (lead).

## Ethics Statement

Ethics approval and consent to participate: Ethical approval for the UK Biobank research was obtained from the North West Multicenter Research Ethics Committee (21/NW/0157), and written informed consent was obtained from all participants prior to their participation in the study.

## Conflicts of Interest

The authors declare no conflicts of interest.

## Supporting information


**Data S1:** fsn370790‐sup‐0001‐supinfo.docx.

## Data Availability

Data are available in a public, open access repository. The UK Biobank data are available on application to the UK Biobank (www.ukbiobank.ac.uk/).
