# Supplementary material for: Association Between Healthful Plant‐Based Dietary Pattern and Adiposity Measures Trajectories and Future Metabolic Diseases Risk: A Prospective Cohort Study
Source: Food Sci Nutr. 2025 Aug 17;13(8):e70790. doi: 10.1002/fsn3.70790 (PMC12358011; doi:10.1002/fsn3.70790)
Supplement: Supplementary file 1 — Data S1: fsn370790‐sup‐0001‐supinfo.docx. [file FSN3-13-e70790-s001.docx]

## Methods

### Adiposity measures trajectories assessment

Distinct trajectories of BMI, WHR or FMI were identified using a latent class mixed modeling (LCMM) approach, which utilized adiposity data from the four time points. This approach assumes that the study population comprises various groups characterized by unique developmental trajectories(Lennon et al., 2018). The analysis was conducted using the "lcmm" package within the R software environment(Proust-Lima, Philipps, & Liquet, 2017). Initially, models were fitted with different numbers of trajectory groups, and the optimal number of trajectory groups was determined based on the Bayesian Information Criterion (BIC). A model was deemed satisfactory if it exhibited an average posterior probability (APP) exceeding 70% and if each trajectory group comprised more than 5% of the total members.

### Dietary assessment and calculation of Plant-Based Diet Indices

Dietary data were obtained through a 24-hour dietary recall questionnaire(Liu et al., 2011). The questionnaire's specifics have been previously outlined and validated for estimating similar nutrient intakes based on a single day's dietary intake(Piernas et al., 2021). We derived the plant-based diet index (PDI), healthful plant-based diet index (hPDI), and unhealthful plant-based diet index (uPDI) by assigning scores to 17 food categories, following established methods(Thompson et al., 2023).

In the PDI, positive scores were assigned to healthy plant foods (such as whole grains, fruits, vegetables, nuts, legumes, vegetarian protein alternatives, tea, and coffee) and unhealthy plant foods (including fruit juices, refined grains, potatoes, sugar-sweetened beverages, sweets, and desserts), while animal foods received reverse scores. For the hPDI, positive scores were assigned to healthy plant foods, while unhealthy plant foods and animal foods received reverse scores. Conversely, in the uPDI, positive scores were assigned to unhealthy plant foods, while healthy plant foods and animal foods received reverse scores.

The 17 food groups, with an intake above 0 portions, were divided into quartiles. Participants received scores ranging from 1 to 5 based on their intake quartile, with a score of 1 assigned to those with no intake and scores ranging from 2 to 5 assigned to quartiles from lowest to highest intake for positive scores. Conversely, participants received scores ranging from 5 to 1 for reverse scores, with a score of 5 assigned to no intake and scores ranging from 1 to 4 assigned to quartiles from highest to lowest intake.

The final PDI, hPDI, and uPDI scores for each participant were calculated by summing the scores from each of the 17 food groups. Subsequently, PDI, hPDI, and uPDI were categorized into three groups: low (<P25), medium (P25-P75), and high (≥P75).


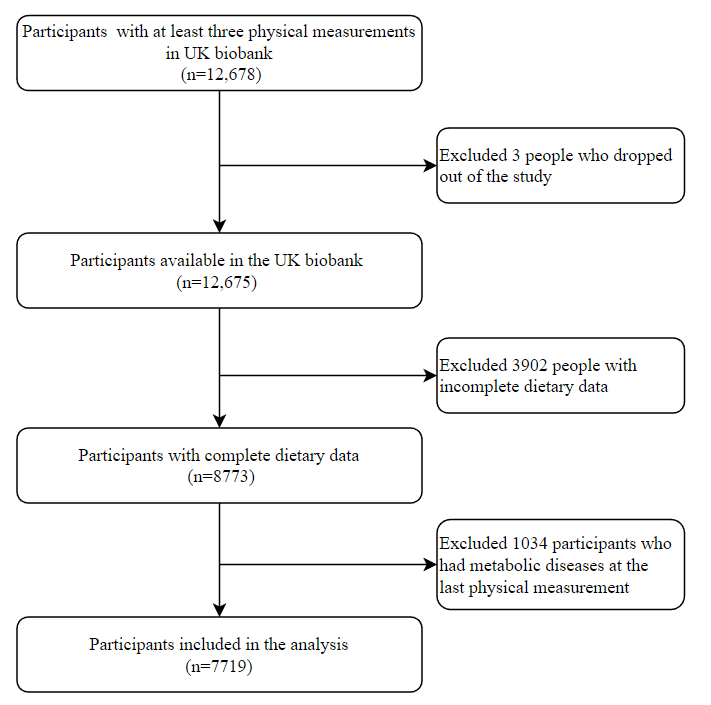


Figure S1. Flow chart for inclusion and exclusion of research subjects

Table S1. ICD-10 codes of metabolic diseases

| Metabolic diseases | Corresponding ICD-10 codes |
| --- | --- |
| Nonalcoholic fatty liver disease | K740, K746, K758, K760, K769 |
| Diabetes | E10-14 |
| Metabolic disorders | E70-79, E80, E83-89 |

Table S2. Characteristics of participants in trajectory groups

| Variables | BMI trajectories | |  | WHR trajectories | |  | FMI trajectories | |
| --- | --- | --- | --- | --- | --- | --- | --- | --- |
|  | Low-Smooth  (n=6823) | High-Growth-Decline  (n=896) |  | Low-Growth  (n=2217) | High-Growth  (n=5502) |  | Low-Smooth  (n=6368) | High-Growth-Decline  (n=1351) |
| Age (mean (SD)) | 54.84 (7.48) | 53.75 (7.46) |  | 54.62 (7.20) | 54.75 (7.60) |  | 54.91 (7.46) | 53.77 (7.52) |
| Sex (%) |  |  |  |  |  |  |  |  |
| Female | 3528 (51.7) | 498 (55.6) |  | 2144 (96.7) | 1882 (34.2) |  | 2890 (45.4) | 1136 (84.1) |
| Male | 3295 (48.3) | 398 (44.4) |  | 73 (3.3) | 3620 (65.8) |  | 3478 (54.6) | 215 (15.9) |
| Ethnic (%) |  |  |  |  |  |  |  |  |
| White | 6651 (97.5) | 872 (97.3) |  | 2176 (98.2) | 5347 (97.2) |  | 6205 (97.4) | 1318 (97.6) |
| Others | 162 (2.4) | 21 (2.3) |  | 41 (1.8) | 142 (2.6) |  | 152 (2.4) | 31 (2.3) |
| Missing | 10 (0.1) | 3 (0.3) |  | 0 (0.0) | 13 (0.2) |  | 11 (0.2) | 2 (0.1) |
| Qualifications (%) |  |  |  |  |  |  |  |  |
| College or University degree | 3599 (52.7) | 403 (45.0) |  | 1176 (53.0) | 2826 (51.4) |  | 3396 (53.3) | 606 (44.9) |
| A levels/AS levels or equivalent | 942 (13.8) | 147 (16.4) |  | 323 (14.6) | 766 (13.9) |  | 871 (13.7) | 218 (16.1) |
| O levels/GCSEs or equivalent | 1201 (17.6) | 171 (19.1) |  | 409 (18.4) | 963 (17.5) |  | 1098 (17.2) | 274 (20.3) |
| Professional qualifications | 307 (4.5) | 39 (4.4) |  | 115 (5.2) | 231 (4.2) |  | 273 (4.3) | 73 (5.4) |
| None of the above | 760 (11.1) | 135 (15.1) |  | 189 (8.5) | 706 (12.8) |  | 718 (11.3) | 177 (13.1) |
| Missing | 14 (0.2) | 1 (0.1) |  | 5 (0.2) | 10 (0.2) |  | 12 (0.2) | 3 (0.2) |
| TDI (mean (SD)) | -2.20 (2.53) | -1.77 (2.77) |  | -2.29 (2.50) | -2.10 (2.58) |  | -2.24 (2.51) | -1.73 (2.74) |
| Smoking (%) |  |  |  |  |  |  |  |  |
| Never | 4326 (63.4) | 515 (57.5) |  | 1530 (69.0) | 3311 (60.2) |  | 4022 (63.2) | 819 (60.6) |
| Previous | 2151 (31.5) | 331 (36.9) |  | 609 (27.5) | 1873 (34.0) |  | 2019 (31.7) | 463 (34.3) |
| Current | 338 (5.0) | 47 (5.2) |  | 77 (3.5) | 308 (5.6) |  | 319 (5.0) | 66 (4.9) |
| Missing | 8 (0.1) | 3 (0.3) |  | 1 (0.0) | 10 (0.2) |  | 8 (0.1) | 3 (0.2) |
| Drinking (%) |  |  |  |  |  |  |  |  |
| Never | 177 (2.6) | 24 (2.7) |  | 70 (3.2) | 131 (2.4) |  | 159 (2.5) | 42 (3.1) |
| Previous | 121 (1.8) | 23 (2.6) |  | 41 (1.8) | 103 (1.9) |  | 115 (1.8) | 29 (2.1) |
| Current | 6525 (95.6) | 848 (94.6) |  | 2106 (95.0) | 5267 (95.7) |  | 6094 (95.7) | 1279 (94.7) |
| Missing | 0 (0.0) | 1 (0.1) |  | 0 (0.0) | 1 (0.0) |  | 0 (0.0) | 1 (0.1) |
| Physical activity (%) |  |  |  |  |  |  |  |  |
| Low | 985 (14.4) | 197 (22.0) |  | 264 (11.9) | 918 (16.7) |  | 911 (14.3) | 271 (20.1) |
| Moderate | 2485 (36.4) | 312 (34.8) |  | 787 (35.5) | 2010 (36.5) |  | 2292 (36.0) | 505 (37.4) |
| High | 2428 (35.6) | 240 (26.8) |  | 769 (34.7) | 1899 (34.5) |  | 2331 (36.6) | 337 (24.9) |
| Missing | 925 (13.6) | 147 (16.4) |  | 397 (17.9) | 675 (12.3) |  | 834 (13.1) | 238 (17.6) |
| PDI (mean (SD)) | 54.82 (4.67) | 54.31 (4.85) |  | 55.16 (4.64) | 54.60 (4.71) |  | 54.79 (4.67) | 54.63 (4.80) |
| hPDI (mean (SD)) | 49.92 (5.48) | 48.46 (5.43) |  | 51.34 (5.25) | 49.11 (5.46) |  | 49.83 (5.48) | 49.35 (5.54) |
| uPDI (mean (SD)) | 49.44 (5.30) | 49.88 (5.60) |  | 49.06 (5.02) | 49.67 (5.45) |  | 49.44 (5.30) | 49.75 (5.50) |
| Metabolic diseases (%) |  |  |  |  |  |  |  |  |
| No | 6410 (93.9) | 797 (89.0) |  | 2117 (95.5) | 5090 (92.5) |  | 5961 (93.6) | 1246 (92.2) |
| Yes | 413 (6.1) | 99 (11.0) |  | 100 (4.5) | 412 (7.5) |  | 407 (6.4) | 105 (7.8) |
| NAFLD (%) |  |  |  |  |  |  |  |  |
| No | 6792 (99.5) | 884 (98.7) |  | 2212 (99.8) | 5464 (99.3) |  | 6339 (99.5) | 1337 (99.0) |
| Yes | 31 (0.5) | 12 (1.3) |  | 5 (0.2) | 38 (0.7) |  | 29 (0.5) | 14 (1.0) |
| Diabetes (%) |  |  |  |  |  |  |  |  |
| No | 6773 (99.3) | 856 (95.5) |  | 2209 (99.6) | 5420 (98.5) |  | 6313 (99.1) | 1316 (97.4) |
| Yes | 50 (0.7) | 40 (4.5) |  | 8 (0.4) | 82 (1.5) |  | 55 (0.9) | 35 (2.6) |
| Metabolic disorders (%) |  |  |  |  |  |  |  |  |
| No | 6457 (94.6) | 828 (92.4) |  | 2124 (95.8) | 5161 (93.8) |  | 6013 (94.4) | 1272 (94.2) |
| Yes | 366 (5.4) | 68 (7.6) |  | 93 (4.2) | 341 (6.2) |  | 355 (5.6) | 79 (5.8) |

Abbreviations: BMI, body mass index; WHR, waist-to-hip ratio; FMI, fat mass index; TDI, Townsend deprivation index; NAFLD, Nonalcoholic fatty liver disease; PDI, plant-based diet index; hPDI, healthful plant-based diet index; uPDI, unhealthful plant-based diet index.


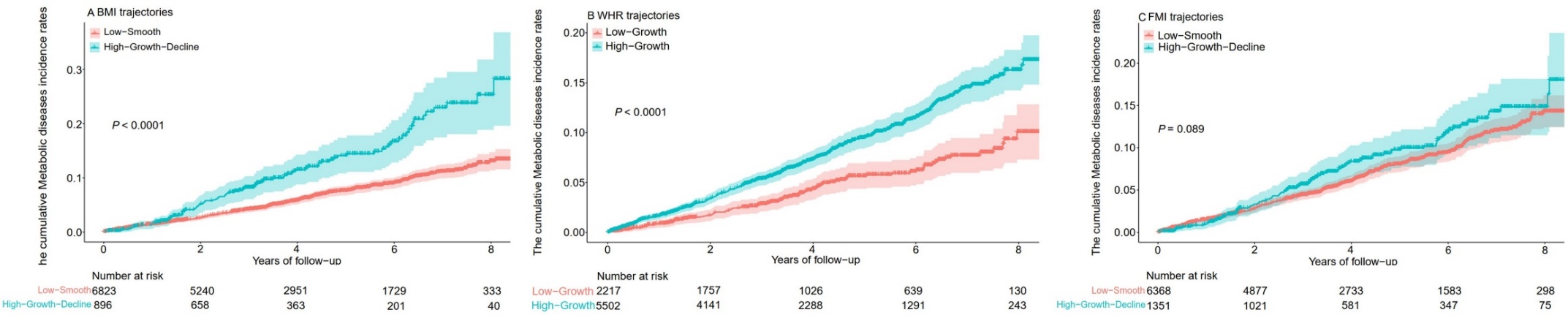


Figure S2. Kaplan–Meier estimates of the cumulative incidence of metabolic diseases by BMI (A), WHR (B) and FMI (C) trajectory groups.

Table S3. Sensitivity analyses 1 (exclusion of participants with less than 2 years of follow-up)

|  | BMI trajectories HR (95% CI) | |  | WHR trajectories HR (95% CI) | |  | FMI trajectories HR (95% CI) | |
| --- | --- | --- | --- | --- | --- | --- | --- | --- |
|  | Low-Smooth  (n=5240) | High-Growth-Decline  (n=658) |  | Low-Growth  (n=1757) | High-Growth  (n=4141) |  | Low-Smooth  (n=4877) | High-Growth-Decline  (n=1021) |
| Metabolic diseases |  |  |  |  |  |  |  |  |
| Cases, n (%) | 254 (4.85) | 60 (9.12) |  | 66 (3.76) | 248 (5.99) |  | 248 (5.09) | 66 (6.46) |
| Model 1 | Ref | 1.936 (1.461, 2.565) |  | Ref | 1.700 (1.296, 2.231) |  | Ref | 1.237 (0.943, 1.623) |
| Model 2 | Ref | 1.963 (1.476, 2.610) |  | Ref | 1.213 (0.862, 1.706) |  | Ref | 1.643 (1.219, 2.214) |
| NAFLD |  |  |  |  |  |  |  |  |
| Cases, n (%) | 17 (0.32) | 11 (1.67) |  | 3 (0.17) | 25 (0.60) |  | 16 (0.33) | 12 (1.18) |
| Model 1 | Ref | 5.200 (2.436, 11.103) |  | Ref | 3.705 (1.119, 12.267) |  | Ref | 3.467 (1.640, 7.330) |
| Model 2 | Ref | 5.278 (2.431, 11.462) |  | Ref | 3.572 (0.959, 13.306) |  | Ref | 5.371 (2.291, 12.59) |
| Diabetes |  |  |  |  |  |  |  |  |
| Cases, n (%) | 30 (0.57) | 22 (3.34) |  | 5 (0.28) | 47 (1.13) |  | 34 (0.70) | 18 (1.76) |
| Model 1 | Ref | 5.920 (3.415, 10.262) |  | Ref | 4.164 (1.656, 10.471) |  | Ref | 2.455 (1.387, 4.348) |
| Model 2 | Ref | 5.976 (3.400, 10.504) |  | Ref | 1.911 (0.634, 5.761) |  | Ref | 5.283 (2.783, 10.028) |
| Metabolic disorders |  |  |  |  |  |  |  |  |
| Cases, n (%) | 225 (4.29) | 38 (5.78) |  | 61 (3.47) | 202 (4.88) |  | 215 (4.41) | 48 (4.70) |
| Model 1 | Ref | 1.366 (0.969, 1.927) |  | Ref | 1.492 (1.121, 1.987) |  | Ref | 1.033 (0.755, 1.412) |
| Model 2 | Ref | 1.391 (0.983, 1.968) |  | Ref | 1.051 (0.727, 1.518) |  | Ref | 1.306 (0.930, 1.834) |

Model 1 had no adjustment covariates; Model 2 was adjusted for age, sex, ethnicity, Townsend deprivation index, educational level, smoking, drinking and physical activity. HR, hazard ratio; CI, Confidence interval; BMI, body mass index; WHR, waist-to-hip ratio; FMI, fat mass index; NAFLD, Nonalcoholic fatty liver disease.

Table S4. Sensitivity analyses 2 (Fine–Gray competing-risk model for adiposity measures trajectories and metabolic diseases)

|  | BMI trajectories HR (95% CI) | |  | WHR trajectories HR (95% CI) | |  | FMI trajectories HR (95% CI) | |
| --- | --- | --- | --- | --- | --- | --- | --- | --- |
|  | Low-Smooth  (n=6823) | High-Growth-Decline  (n=896) |  | Low-Growth  (n=2217) | High-Growth  (n=5502) |  | Low-Smooth  (n=6368) | High-Growth-Decline  (n=1351) |
| Metabolic diseases |  |  |  |  |  |  |  |  |
| Cases, n (%) | 413 (6.05) | 99 (11.05) |  | 100 (4.51) | 412 (7.49) |  | 407 (6.39) | 105 (7.77) |
| Death, n (%) | 99 (1.45) | 22 (2.46) |  | 25 (1.13) | 96 (1.74) |  | 100 (1.57) | 21 (1.55) |
| Model 1 | Ref | 1.902 (1.529, 2.367) |  | Ref | 1.788 (1.437, 2.224) |  | Ref | 1.206 (0.973, 1.494) |
| Model 2 | Ref | 1.910 (1.531, 2.383) |  | Ref | 1.314 (1.004, 1.720) |  | Ref | 1.555 (1.225, 1.973) |
| NAFLD |  |  |  |  |  |  |  |  |
| Cases, n (%) | 31 (0.45) | 12 (1.34) |  | 5 (0.23) | 38 (0.69) |  | 29 (0.46) | 14 (1.04) |
| Death, n (%) | 99 (1.45) | 22 (2.46) |  | 25 (1.13) | 96 (1.74) |  | 100 (1.57) | 21 (1.55) |
| Model 1 | Ref | 3.003 (1.544, 5.842) |  | Ref | 3.209 (1.264, 8.143) |  | Ref | 2.250 (1.191, 4.251) |
| Model 2 | Ref | 2.997 (1.524, 5.890) |  | Ref | 3.050 (1.113, 8.353) |  | Ref | 3.099 (1.510, 6.363) |
| Diabetes |  |  |  |  |  |  |  |  |
| Cases, n (%) | 50 (0.73) | 40 (4.46) |  | 8 (0.36) | 82 (1.49) |  | 55 (0.86) | 35 (2.59) |
| Death, n (%) | 99 (1.45) | 22 (2.46) |  | 25 (1.13) | 96 (1.74) |  | 100 (1.57) | 21 (1.55) |
| Model 1 | Ref | 6.307 (4.161, 9.560) |  | Ref | 4.338 (2.098, 8.971) |  | Ref | 2.990 (1.956, 4.572) |
| Model 2 | Ref | 6.039 (3.989, 9.144) |  | Ref | 3.035 (1.278, 7.206) |  | Ref | 4.979 (3.042, 8.150) |
| Metabolic disorders |  |  |  |  |  |  |  |  |
| Cases, n (%) | 366 (5.36) | 68 (7.59) |  | 93 (4.19) | 341 (6.20) |  | 355 (5.57) | 79 (5.85) |
| Death, n (%) | 99 (1.45) | 22 (2.46) |  | 25 (1.13) | 96 (1.74) |  | 100 (1.57) | 21 (1.55) |
| Model 1 | Ref | 1.453 (1.122, 1.881) |  | Ref | 1.580 (1.257, 1.987) |  | Ref | 1.033 (0.809, 1.317) |
| Model 2 | Ref | 1.465 (1.129, 1.902) |  | Ref | 1.139 (0.855, 1.517) |  | Ref | 1.287 (0.985, 1.683) |

Model 1 had no adjustment covariates; Model 2 was adjusted for age, sex, ethnicity, Townsend deprivation index, educational level, smoking, drinking and physical activity. HR, hazard ratio; CI, Confidence interval; BMI, body mass index; WHR, waist-to-hip ratio; FMI, fat mass index; NAFLD, Nonalcoholic fatty liver disease.


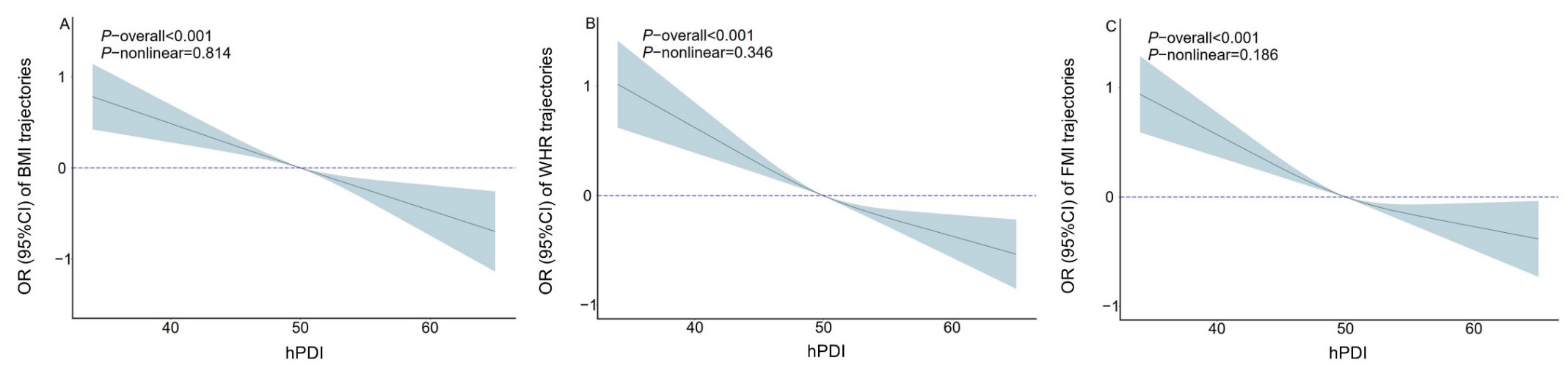


Figure S3. Dose–response associations of hPDI scores and the High-Growth-Decline BMI (A), High-Growth WHR (B), and High-Growth-Decline FMI trajectories (C). Restricted cubic spline regression adjusted for age, sex, ethnic, Townsend deprivation index, qualifications, smoking, drinking and physical activity.

Table S5. Sensitivity analyses 3 (exclusion of participants with missing covariates)

|  | BMI trajectories HR (95% CI) | |  | WHR trajectories HR (95% CI) | |  | FMI trajectories HR (95% CI) | |
| --- | --- | --- | --- | --- | --- | --- | --- | --- |
|  | Low-Smooth  (n=6823) | High-Growth-Decline  (n=896) |  | Low-Growth  (n=2217) | High-Growth  (n=5502) |  | Low-Smooth  (n=6368) | High-Growth-Decline  (n=1351) |
| Metabolic diseases |  |  |  |  |  |  |  |  |
| Cases, n (%) | 413 (6.05) | 99 (11.05) |  | 100 (4.51) | 412 (7.49) |  | 407 (6.39) | 105 (7.77) |
| Model 1 | Ref | 1.916 (1.498, 2.452) |  | Ref | 1.943 (1.507, 2.506) |  | Ref | 1.255 (0.988, 1.594) |
| Model 2 | Ref | 1.955 (1.522, 2.511) |  | Ref | 1.458 (1.070, 1.987) |  | Ref | 1.734 (1.331, 2.259) |
| NAFLD |  |  |  |  |  |  |  |  |
| Cases, n (%) | 31 (0.45) | 12 (1.34) |  | 5 (0.23) | 38 (0.69) |  | 29 (0.46) | 14 (1.04) |
| Model 1 | Ref | 2.903 (1.410, 5.977) |  | Ref | 3.380 (1.199, 9.522) |  | Ref | 2.273 (1.147, 4.505) |
| Model 2 | Ref | 2.973 (1.424, 6.207) |  | Ref | 2.912 (0.913, 9.286) |  | Ref | 3.626 (1.668, 7.883) |
| Diabetes |  |  |  |  |  |  |  |  |
| Cases, n (%) | 50 (0.73) | 40 (4.46) |  | 8 (0.36) | 82 (1.49) |  | 55 (0.86) | 35 (2.59) |
| Model 1 | Ref | 6.928 (4.328, 11.088) |  | Ref | 5.173 (2.083, 12.847) |  | Ref | 3.307 (2.050, 5.335) |
| Model 2 | Ref | 6.888 (4.245, 11.179) |  | Ref | 4.180 (1.554, 11.246) |  | Ref | 5.859 (3.382, 10.151) |
| Metabolic disorders |  |  |  |  |  |  |  |  |
| Cases, n (%) | 366 (5.36) | 68 (7.59) |  | 93 (4.19) | 341 (6.20) |  | 355 (5.57) | 79 (5.85) |
| Model 1 | Ref | 1.407 (1.045, 1.894) |  | Ref | 1.730 (1.323, 2.262) |  | Ref | 1.047 (0.794, 1.380) |
| Model 2 | Ref | 1.433 (1.061, 1.937) |  | Ref | 1.295 (0.929, 1.804) |  | Ref | 1.375 (1.017, 1.859) |

Model 1 had no adjustment covariates; Model 2 was adjusted for age, sex, ethnicity, Townsend deprivation index, educational level, smoking, drinking and physical activity. HR, hazard ratio; CI, Confidence interval; BMI, body mass index; WHR, waist-to-hip ratio; FMI, fat mass index; NAFLD, Nonalcoholic fatty liver disease.

Table S6. Logistic regression models for plant-based dietary patterns and adiposity measures trajectories in participants excluding missing covariates

| Diet index | High-Growth-Decline BMI trajectory |  | High-Growth WHR trajectory |  | FMI High-Growth-Decline trajectory |
| --- | --- | --- | --- | --- | --- |
|  | OR (95% CI) |  | OR (95% CI) |  | OR (95% CI) |
| Group of PDI |  |  |  |  |  |
| Low | Ref |  | Ref |  | Ref |
| Medium | 0.786 (0.653, 0.945) |  | 0.953 (0.803, 1.131) |  | 0.883 (0.744, 1.048) |
| High | 0.777 (0.629, 0.960) |  | 0.938 (0.776, 1.133) |  | 0.855 (0.705, 1.036) |
| Group of hPDI |  |  |  |  |  |
| Low | Ref |  | Ref |  | Ref |
| Medium | 0.680 (0.566, 0.817) |  | 0.687 (0.567, 0.832) |  | 0.689 (0.577, 0.823) |
| High | 0.487 (0.391, 0.607) |  | 0.528 (0.432, 0.644) |  | 0.500 (0.410, 0.609) |
| Group of uPDI |  |  |  |  |  |
| Low | Ref |  | Ref |  | Ref |
| Medium | 1.003 (0.821, 1.224) |  | 1.054 (0.892, 1.247) |  | 1.005 (0.841, 1.200) |
| High | 1.035 (0.831, 1.290) |  | 1.302 (1.075, 1.577) |  | 1.174 (0.964, 1.429) |

Logistic regression model was adjusted for age, sex, ethnicity, Townsend deprivation index, educational level, smoking, drinking and physical activity. OR, odds ratio; CI, confidence interval; SD, standard deviation; BMI, body mass index; WHR, waist-to-hip ratio; FMI, fat mass index; PDI, plant-based diet index; hPDI, healthful plant-based diet index; uPDI, unhealthful plant-based diet index.

## References

Lennon, H., Kelly, S., Sperrin, M., Buchan, I., Cross, A. J., Leitzmann, M., . . . Renehan, A. G. (2018). Framework to construct and interpret latent class trajectory modelling. *BMJ Open, 8*(7). doi:10.1136/bmjopen-2017-020683

Liu, B., Young, H., Crowe, F. L., Benson, V. S., Spencer, E. A., Key, T. J., . . . Beral, V. (2011). Development and evaluation of the Oxford WebQ, a low-cost, web-based method for assessment of previous 24 h dietary intakes in large-scale prospective studies. *Public Health Nutrition, 14*(11), 1998-2005. doi:10.1017/S1368980011000942

Piernas, C., Perez-Cornago, A., Gao, M., Young, H., Pollard, Z., Mulligan, A., . . . Jebb, S. A. (2021). Describing a new food group classification system for UK biobank: analysis of food groups and sources of macro- and micronutrients in 208,200 participants. *European Journal of Nutrition, 60*(5), 2879-2890. doi:10.1007/s00394-021-02535-x

Proust-Lima, C., Philipps, V., & Liquet, B. (2017). Estimation of Extended Mixed Models Using Latent Classes and Latent Processes: The R Package lcmm. *Journal of Statistical Software, 78*(2). doi:10.18637/jss.v078.i02

Thompson, A. S., Tresserra-Rimbau, A., Karavasiloglou, N., Jennings, A., Cantwell, M., Hill, C., . . . Kühn, T. (2023). Association of Healthful Plant-based Diet Adherence With Risk of Mortality and Major Chronic Diseases Among Adults in the UK. *JAMA Network Open, 6*(3), e234714. doi:10.1001/jamanetworkopen.2023.4714
